# Designs, applications, and limitations of genetically encoded fluorescent sensors to explore plant biology

**DOI:** 10.1093/plphys/kiab353

**Published:** 2021-08-30

**Authors:** Mayuri Sadoine, Yuuma Ishikawa, Thomas J. Kleist, Michael M. Wudick, Masayoshi Nakamura, Guido Grossmann, Wolf B. Frommer, Cheng-Hsun Ho

**Affiliations:** 1 Molecular Physiology, Heinrich-Heine-University Düsseldorf, Düsseldorf 40225, Germany; 2 Institute of Transformative Bio-Molecules (WPI-ITbM), Nagoya University, Chikusa, Nagoya 464-8601, Japan; 3 Cluster of Excellence on Plant Sciences, Heinrich-Heine-University Düsseldorf, Düsseldorf 40225, Germany; 4 Institute for Cell and Interaction Biology, Heinrich-Heine-University Düsseldorf, Düsseldorf 40225, Germany; 5 Agricultural Biotechnology Research Center, Academia Sinica, Taipei 115, Taiwan

## Abstract

The understanding of signaling and metabolic processes in multicellular organisms requires knowledge of the spatial dynamics of small molecules and the activities of enzymes, transporters, and other proteins in vivo, as well as biophysical parameters inside cells and across tissues. The cellular distribution of receptors, ligands, and activation state must be integrated with information about the cellular distribution of metabolites in relation to metabolic fluxes and signaling dynamics in order to achieve the promise of in vivo biochemistry. Genetically encoded sensors are engineered fluorescent proteins that have been developed for a wide range of small molecules, such as ions and metabolites, or to report biophysical processes, such as transmembrane voltage or tension. First steps have been taken to monitor the activity of transporters in vivo. Advancements in imaging technologies and specimen handling and stimulation have enabled researchers in plant sciences to implement sensor technologies in intact plants. Here, we provide a brief history of the development of genetically encoded sensors and an overview of the types of sensors available for quantifying and visualizing ion and metabolite distribution and dynamics. We further discuss the pros and cons of specific sensor designs, imaging systems, and sample manipulations, provide advice on the choice of technology, and give an outlook into future developments.

## Teaser

Different types of genetically encoded sensors in plants can be used to quantify and visualize ion and metabolite distributions and dynamics.

## Introduction

Most physiological models are based on data from a combination of in vitro biochemistry, that is, the biochemical characterization of enzymes and transporters in vitro or in heterologous systems, and steady-state metabolite level analysis. Quantitation of metabolite levels typically involves extraction from whole organs, thus averaging not only across different cell types but also compartments. Single-cell sequencing has advanced our perspectives by providing insights into the cellular transcriptomes, enabling analyses on cell type-specific pathway activities (Kim et al., 2021). However, transcript levels of individual genes are not necessarily correlated with respective protein levels, and single-cell proteomics and metabolomics are not yet at a level that would offer sufficient resolution and depth (Walley et al., 2016; Marx, 2019). Moreover, enzymes and transporters handle multiple substrates and their activities are finely tuned by posttranslational modifications and allosteric effectors. To elucidate cell type-specific pathway dynamics in plants, we need in vivo biochemistry tools that quantify metabolites, fluxes, and activities in planta.

**Advances**

 Various tools have been developed to measure the distribution of small molecules with cellular resolution. Genetically encoded sensors have emerged that can provide not only quantitative information about ion and metabolite dynamics but can also be used to monitor the activity of proteins in vivo with comparatively low invasiveness. Over the past two decades, progress in sensor engineering and fluorescence imaging has led to a broad range of tools for monitoring small molecules and a series of activity sensors. Importantly, genetically encoded sensors can be targeted to specific cellular or subcellular locations or even fused to specific proteins such as transporters to obtain unprecedented spatial resolution. Such sensors can detect changes in the concentration and distribution of small molecules in living tissues at rates down to the Hertz range (60 s^−1^), which has rendered them the primary means of recording molecular dynamics in living cells. Here, we summarize progress of the recent years and available tools. We focus on sensors that make use of fluorescent proteins (FPs) as reporter elements. An overview over the repertoire of sensors available for ions, metabolites, hormones, and transporter activity relevant for plant science can be found in several fluorescent sensor databases (www.molecular-physiology.hhu.de/resources; https://biosensordb.ucsd.edu/index.php). In addition, we provide an update on tools available to quantitative fluorescence imaging of sensor output.

- Over the past decades, a wide range of sensors including actvity sensors were successfully engineered for ions, metabolites, and hormones.
- High-throughput screening of sensor candidates will be key to rapid development of ultrasensitive sensors.
- The discovery of the circular permutation concept was a breakthrough for the design of ultrasensitive sensors and, combined with reference FPs, provides ultrasensitive ratiometric sensors.
- Perfusion and microfluidic systems have been key technologies for discoveries using sensors.
- Advanced imaging systems and associated technologies have been critical for dealing with obstacles of dimensionality and autofluorescence in plant.

## A short history of genetically encoded sensors

**Figure 1:**
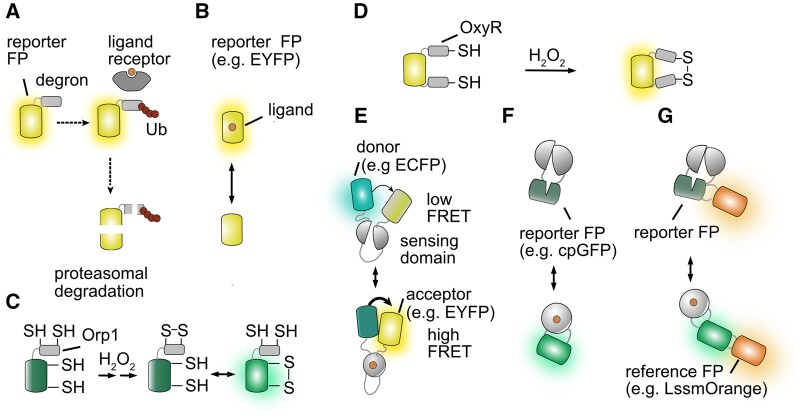
Genetically encoded sensors. A, Degron-FP fusion; (B) FP as recognition element; (C) roGFP2-Orp1 and (D) HyPer sensors; (E) ratiometric FRET-based sensors consisting of two FPs and a recognition element; (F) intensiometric cpFP-recognition element fusion; and (G) ratiometric Matryoshka sensor with cpFP and nested reference FP.

Before the advent of genetically encoded sensors, organic fluorescent dyes that change optical properties in the presence of specific ligands were used to monitor calcium spiking in cell cultures, for example, calcium dyes such as FURA-2 or FlAsH, or the elegant Förster resonance energy transfer (FRET)-based voltage dyes developed by the late Roger Tsien (Tsien, 1980; Grynkiewicz et al., 1985). The dyes enabled many discoveries; however, microinjection was required and dyes could not be targeted to specific cell types or subcellular compartments. The discovery of the FP from jellyfish, namely green FP (GFP), opened the door for engineering genetically encoded sensors (Shimomura et al., 1962; Chalfie et al., 1994; Tsien, 1998; Frommer et al., 2009). Roger Tsien built on the FRET concept by engineering spectral FP variants that made them suitable for FRET (Heim and Tsien, 1996). The breakthrough idea behind the design of genetically encoded, fluorescence-based sensors was to use analyte-induced conformational rearrangements in a protein domain that binds an analyte and fusion to FRETable FP variants. Analyte-binding-induced conformational rearrangements lead to a change in the relative emission of the two FPs upon donor excitation. The ratio change was proportional to the change in ligand concentration, following the binding isotherm of the recognition element. Thus here, protein conformation is used as a proxy for the analyte concentration. Miyawaki and Tsien developed cameleon as the prototype, by combining massive conformational rearrangements in calmodulin and further enhancement by a calmodulin binding domain that binds to the calcium-bound form of calmodulin (Miyawaki et al., 1997). Notably, a similar sensor just using calmodulin as a recognition element was published in the same year (Romoser et al., 1997). About 25 years of engineering and optimization of FPs and linkers were required to achieve levels of signal-to-noise ratio (SNR) and sensitivity sufficient, resulting in substantially improved sensors for in vivo imaging (Allen et al., 2001; Deuschle et al., 2006; Chaudhuri et al., 2008). Since ratio changes can be triggered by other factors, for example, differential bleaching, fluorescence lifetime (FL) measurements provide a more rigid way of quantifying actual FRET changes. Yet, some of the best sensors use a different mechanism: Again, Tsien’s lab laid the basis by engineering a conformationally sensitive circular permutated GFP (cpGFP) and used it to generate intensiometric sensors with exceptional sensitivity (Baird et al., 1999). In the following section we will discuss different sensor types and different read out options (FRET sensors, intensiometric single FP sensors, ratiometric single FP sensors with a reference FP, FL sensors, and photoacoustic sensors). We then turn to activity sensors before describing applications of sensors for ions and metabolites in plants. Degron-based reporters, that make use of ligand-induced degradation of the FP fused to a degron will not be discussed here (Figure 1A; Santner and Estelle, 2010). SNAP-tagging of chemical sensors is discussed in Box 1 (Keppler et al., 2003; Tomat et al., 2008; Kamiya and Johnsson, 2010; Carter et al., 2014; Jiao et al., 2018; Iwatate et al., 2020).

Box 1. Alternatives: SNAP-tagging of chemical sensors.

A wide variety of fluorescent chemical probes have been developed for sensing and imaging the presence of specific ligands in the biological systems (Carter et al., 2014; Jiao et al., 2018). Covalent self-labeling technologies such as SNAP-tag, which localizes synthetic chemical dyes to specific proteins, were pioneered by Johnsson’s group (Keppler et al., 2003), allowing the targeting of fluorescent chemical probes to specific subcellular domains and proteins. SNAP-tag is a human DNA repair enzyme O^6^-alkylguanine transferase genetically fused to the target protein, which reacts with a benzylguanine-conjugated fluorescent dye to form a covalent bond that labels explicitly the protein. In mammalian cells, the fluorescent small molecule zinc sensor Zinpyr (ZP1) has been successfully targeted to mitochondria and Golgi apparatus using SNAP-tag to quantitatively visualize a turn-on emission response to addition of zinc (Tomat et al., 2008). Kamiya and Johnsson successfully targeted a BODIPY-based calcium indicator to a specific compartment using SNAP-tag to measure local calcium concentration (Kamiya and Johnsson, 2010). In principle, this approach is generalizable to any fluorescent chemical probes that can be modified with O^6^-benzylguanine moieties. Recently, our group has demonstrated that covalent labeling of SNAP-tag with synthetic chemical dyes occurs in living plant cells and that SNAP-tagging technology is applicable to plant research (Iwatate et al., 2020). SNAP-tagging of chemical dyes is a promising technology that complements and combines the benefits of FP sensors and offers advantages over FP sensors, including the availability of a vast repertoire of designed and synthesized chemical probes.

### FPs as sensory domains and reporters

Direct effects of pH or redox on FPs can be used to construct sensors (Miesenböck et al., 1998; Kuner and Augustine, 2000; Figure 1, B and C). The conformation of FPs and thus emission depend on pH (Chattoraj et al., 1996). Thus, FPs with a pK_a_ close to physiological conditions can be used for pH sensing, for example, pHlourin and ratiometric pHluorin/phGFP, Pt-GFP (Miesenböck et al., 1998; Moseyko and Feldman, 2001; Schulte et al., 2006). The sensitivity to anions was used to constructs chloride and iodide sensors (YFP-H148Q, Clomeleon); ClopHensor reports pH and chloride dynamics (Galietta et al., 2001; Wu et al., 2020). Other similar sensors can be used for measuring redox status, or metal and calcium concentrations (Østergaard et al., 2001; Hanson et al., 2004; Chapleau et al., 2008; Tang et al., 2011). roGFP- and HyPer-type sensors were used to probe redox status and H_2_O_2_, a reactive oxygen species (ROS) in plant stress and physiology (Belousov et al., 2006; Gutscher et al., 2009; Costa et al., 2010; Müller-Schüssele et al., 2021; Figure 1, C and D). RoGFP-based sensors take advantages of modified redox- or ROS-sensitive FPs that are used alone or fused to sensory domains (human Grx1, yeast peroxidase Orp1, modified 2-Cys peroxiredoxin Tsa2; Gutscher et al., 2009; Morgan et al., 2016). HyPer1, 2, 3 contain a circular permutated FP (cpFP) fused to a bacterial H_2_O_2_ sensory domain derived from OxyR, which contains two cysteines. H_2_O_2_ induces disulfide bridge formation triggering conformational rearrangements (Belousov et al., 2006). HyPer7 uses a modified cpYFP as reporter providing reduced pH sensitivity (Pak et al., 2020; Ugalde et al., 2021b). The sensitivity of FPs to environmental conditions marks a general challenge: sensor conformation depends on ionic conditions, pH, redox, and interaction of all components of a sensor with cellular environment, therefore potentially leading to misinterpretation of the underlying cause of the observed response.

### FRET sensors

Tsien’s fundamental concept was used to engineer FRET sensors for a wide range of applications. The Venus flytrap movement induced by ligand binding in bacterial periplasmic binding proteins (PBPs) was successfully used to generate FRET sensors for maltose without the need of response amplifiers as in cameleon (Fehr et al., 2002). The use of a bacterial protein in eukaryotes had the additional benefit that its use reduced the likelihood of interference by interactions with bona fide interactors. PBPs constitute a large family of proteins that specifically bind a diverse set of analytes, from ions to sugars, from amino acids to small peptides. It became rapidly clear that the same strategy could be used to develop a wide range for sensors for all these analytes (Figure 1E;www.molecular-physiology.hhu.de/resources). PBPs are characterized by high native affinities in the nano- to low micromolar range, which seemed unaltered by fusion to FPs. The affinities are, however, likely too high for measurements of these ions and metabolites in vivo. Each sensor has an approximate detection range of two orders of magnitude around the *K_d_*. In essentially all cases, series of affinity mutants were generated that can cover the range between nanomolar and millimolar (Fehr et al., 2002, 2003; Lager et al., 2003; Okumoto et al., 2005; Sadoine et al., 2021). Since physiological analyte concentrations in the compartments are unknown, affinity variants should be tested. Affinity variants differ in one or few side chains in the binding pocket, and it is likely that environmental sensitivity is comparable. Therefore, affinity mutants help to exclude artifacts, for example, due to pH changes. Sensors can be improved by modifying linkers, swapping FPs, or FP insertion into the backbone of recognition elements (Deuschle et al., 2005; Vinkenborg et al., 2007; Kaper et al., 2008; Sadoine et al., 2021). While not a topic here, the concept has been expanded to measure biophysical parameters such as tension, or to visualize cell cycle phase for each cell during gastrulation (Sakaue-Sawano et al., 2008; Grashoff et al., 2010; Sakaue-Sawano and Miyawaki, 2014).

Roger Tsien’s concept has been extended for measuring transporter activity in vivo. A transporter activity sensor for monitoring nitrate uptake was generated by flanking the nitrate transceptor (transporter/receptor) NTR1.1/CHL1 with two FP variants (Ho and Frommer, 2014). Notably, engineering of FRET sensors has been surprisingly easy: simple sandwiching of candidate recognition elements between two fluorophores was frequently successful to yield a first functional sensor. Improving the sensors is more challenging, requiring empirical engineering and screening of large numbers of variants; cell sorting or high-throughput screening can help with rapid optimization (Zhao et al., 2011; Wardill et al., 2013; Litzlbauer et al., 2015; Nadler et al., 2016).

### Intensiometric single-FP sensors

Intensiometric single-FP sensors rely on changes in fluorescence intensity of a single FP, typically cpFP (Nasu et al., 2021; Figure 1F). Circularly permutation moves N- and C-termini to a new position in the β-barrel at the tip of the chromophore and connects the original termini (Baird et al., 1999). Thereby, the chromophore becomes exposed to the medium, resulting in the disruption of excited state proton transfer (ESPT; i.e. the ability to transfers a proton through a hydrogen bond network generating an excited state anion). The fusion of a conformationally sensitive cpFP to a recognition element affects ESPT and thus emission intensity (Romei and Boxer, 2019). In theory, cpFP-based sensors can cover a large intensity range; the quantum yield before and after ligand addition can shift between 0% and 100%. The resulting sensors, thus, exhibit high sensitivity and improved SNRs relative to FRET-based sensors. Ratiometric FRET sensors are usually easier to develop and are better suited for quantitative imaging, which helps mitigating artifacts typically associated with a difference in expression levels. Engineering cpFP-sensors is more challenging, since it requires identification of a “sweet” spot in the recognition element as well as suitable residues at the fusion site that restore ESPT (Akerboom et al., 2009; De Michele et al., 2013). Cell sorting of mutant libraries and transposon insertion were used for the optimization of maltose sensors (Marvin et al., 2011). Machine learning helped in engineering high dynamic range, sensitive, and selective serotonin sensors (Unger et al., 2020). A potential drawback of these sensors is the pH sensitivity of cpFPs, which can lead to artifacts. All sensors described so far make allow quantification of fluorescence intensity. Alternatively, other detection modes can be advantageous, for example, a more direct FRET analysis that measures donor FL or photoacoustic effects.

### FL sensors

The quantification of FL changes has advantages over intensity ratioing. FL-sensors can be multiplexed because FL is recorded only for the donor, in the presence of a dark acceptor (Greenwald et al., 2018). FL microcopy (FLIM) requires suitable instrumentation (Reissaus et al., 2020). For yet unknown reasons, some FRET sensors tested did not show substantial changes in FL. It has been suggested that sensors require optimization for FLIM. FLIM was successfully implemented for the glucose sensor iGlucoSnFR-TS (Díaz‐García et al., 2019).

### Photoacoustic sensors

Optical imaging is limited by photobleaching during repetitive exposure to high light intensities and by light scattering. Optoacoustic (OA) imaging combines advantages of optical and ultrasound imaging (Hofmann et al., 2019). OA imaging uses nonionizing laser pulses, part of which are converted into heat, transient thermoelastic expansion and ultrasonic emission, and is detected by ultrasonic transducers (Hofmann et al., 2019). Broadband ultrasound waves generated by transient light absorption enable high-resolution imaging at centimeter-scale depths. OA imaging has successfully been used in mice expressing GCaMP6f (Gottschalk et al., 2019).

### Matryoshka sensors

While intensiometric single-FP sensors reach exceptionally high SNRs, sensitivity and dynamic range compared to FRET-based sensors, the readout is sensitive to changes in sensor levels. Fusion of a cpFP sensor to a reference fluorophore that is not subject to FRET converts intensiometric into ratiometric sensors (Ast et al., 2017; Waadt et al., 2017). For the Matryoshka technology a large Stokes shift reference FP is nested into cpFP, allowing for simultaneous excitation of both FPs with a single wavelength (Ast et al., 2017; Figure 1G). The large Stokes shift FP (i.e. LSSmOrange) is inserted into the loop created by circular permutation at the “former” N- and C-termini (Ast et al., 2017). LSSmOrange remains insensitive to conformational effects on cpFP as, for example, in the calcium sensor GO-MatryoshCaMP and the ammonium transporter activity sensor AmTryoshka (Ast et al., 2017). Potential drawbacks of the introduction of a second FP include coverage of large parts of the usable spectrum, reducing options for multiplexing, as well as the presence of an additional terminal or the larger “double FP,” which can impair trafficking, that is, when targeting sensors to the plasma membrane.

## Sensors in plant sciences and discoveries

**Figure 2:**
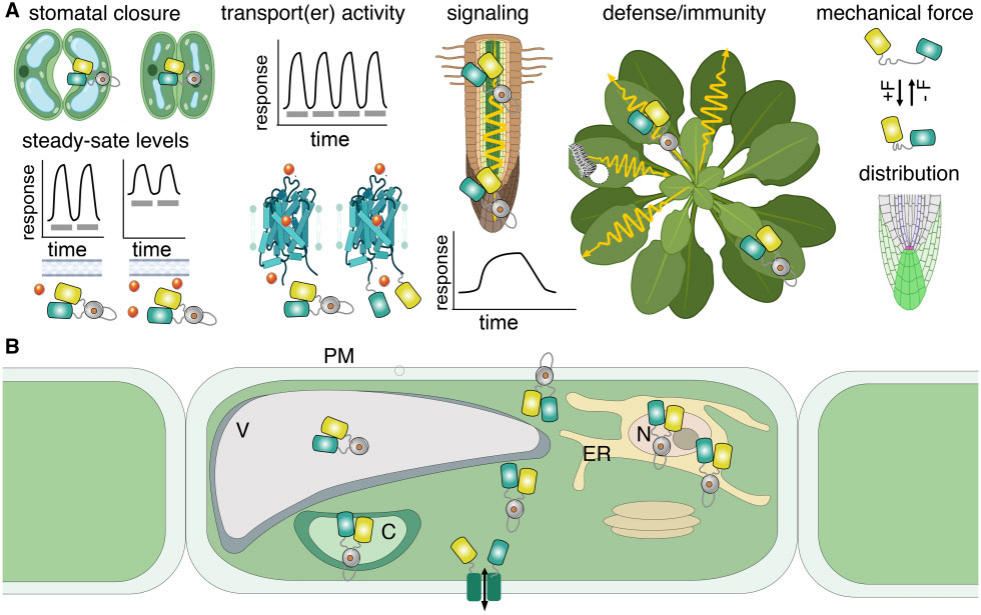
Use of sensors in plant science. A, Analysis of physiological, cellular and molecular processes with the help of genetically encoded sensors (B) targeting of sensors to specific subcellular compartments. PM, plasma membrane; V, vacuole; C, chloroplast; N, nucleus. Figure partially created with BioRender.com.

Soon after Miyawaki and Tsien developed the first calcium sensors, cameleon was used to monitor signaling processes in plant stomata (Allen et al., 2001). Analyses required stably transformed plants, suitable sample preparation, in this case epidermal slices, and imaging technology (Figure 2). After successful engineering of the first metabolite sensors, these were used in Arabidopsis leaves and roots to monitor metabolite dynamics (Deuschle et al., 2006; Chaudhuri et al., 2008). Subsequent progress slowed due to the need for iterative process of sensor improvements, which in the mammalian field is supported by a large number of teams, and is still ongoing even in the field of calcium sensors. Sensors developed for plant or animal biology are readily usable across platforms even in microbes, for example, cAMP sensors work well in yeast (Bermejo et al., 2013), and glucose sensors have successfully been used in bacteria, yeast, plants, and animal cells (Chaudhuri et al., 2008; Takanaga et al., 2008; Bermejo et al., 2010). We hypothesize that with a few exceptions all sensors developed for a particular field can be used across kingdoms. Besides the ability to monitor analyte levels and fluxes, FRET sugar sensors were successfully used to screen mutant populations in yeast to identify regulatory networks controlling glucose uptake, or to identify the elusive sucrose efflux transporters required for phloem loading (Chen et al., 2010, 2012; Bermejo et al., 2013). Researchers in the plant field engineered a large number of different sensors; however, due to the comparatively small community and the wide spectrum of target analytes, many of these sensors still require substantial effort regarding optimization (www.molecular-physiology.hhu.de/resources). For hormone sensing we confer to a recent review (Isoda et al., 2021), and we will not discuss this topic in detail.

### Steady-state levels and net flux rates

Chemically and genetically encoded sensors were originally developed for rapid signaling processes such as calcium spiking. In comparison, dynamic changes for other ions, metabolites, and hormones are orders of magnitude slower. It is important to note that the steady-state level of a compound is determined by multiple rate constants, which typically include the rates of uptake by and efflux from a cell, rates of synthesis and degradation, buffering capacity of the compartments, and rates of import into or release from subcellular compartments (Okumoto et al., 2008). Transport rates can differ dramatically—while in the case of calcium, individual ion channels conduct up to hundreds of millions of units per second, transporters for metabolites move only a handful per second. When measuring analyte levels, one needs to take into account that the rate of uptake may be limiting, and much slower than metabolism, a case in which the analyte levels do not increase. It is generally recommended to use multiple sensors with overlapping detection ranges to explore the accumulation or depletion of analytes and to evaluate possible artifacts.

#### Twenty-five years of calcium sensor optimization and no end in sight

Calcium sensors have been optimized extensively (www.molecular-physiology.hhu.de/resources). Genetically encoded calcium indicators have been reviewed extensively; here, we briefly describe subcellular targeting and implementation in plants (Rose et al., 2014; Walia et al., 2018; Nasu et al., 2021). Cameleon sensors were first used in Arabidopsis guard cells to visualize calcium oscillations (Pei et al., 2000; Ding et al., 2015; Walia et al., 2018). In pollen tubes, cameleon was used to investigate how the calcium-permeable channel cyclic nucleotide-gated channel CNGC18, ROS-producing NADPH oxidases and d-serine affect calcium oscillations (Michard et al., 2011; Lassig et al., 2014). The intensiometric R-GECO1 was used to detect calcium dynamics in leaves in response to treatment with pathogen-associated molecular pattern compounds (Ngo et al., 2014). YC3.6 detected calcium changes during dehydration of *Physcomitrium* (Storti et al., 2018). Major breakthroughs that involved calcium sensors were the identification of salt-induced calcium waves traveling from root tips to the shoot and the discovery of wound-induced systemic calcium waves that depend on plant glutamate receptors (Choi et al., 2014; Toyota et al., 2018). Several studies targeted sensors to subcellular compartments such as chloroplasts, mitochondria, nucleus, peroxisomes, and endoplasmic reticulum (ER; Krebs et al., 2012; Costa and Kudla, 2015). Plastid-targeted sensor analyses indicated that basal calcium concentrations in root plastids are low and recorded stromal calcium signatures (Loro et al., 2016).

#### Sensors for soluble carbohydrates

Fluorescent indicator protein (FLIP) sugar sensors use bacterial PBPs, protein with high affinity to sugars and a well-known “hinge-bend” motion upon binding to maltose, glucose/galactose, arabinose, ribose, sucrose, and trehalose (www.molecular-physiology.hhu.de/resources; Fehr et al., 2002, 2003; Lager et al., 2006; Kaper et al., 2008; Sadoine et al., 2021). The improved FLIPglu-Δ13 glucose family has affinities of 170 nM to 3.2 mM and was used for analyses of cytosolic glucose levels in leaves and roots (Deuschle et al., 2006). The highest affinity sensors (*K_d_* 170 nM, 2 μM) responded to exogenous glucose in roots, but not in leaves, likely due to saturation by cytosolic base levels (Deuschle et al., 2006). FLIPglu-Δ13 glucose sensors also responded to sucrose in roots, indicative of sucrose hydrolysis (Chaudhuri et al., 2008). The sucrose sensor FLIPsuc-90µΔ1 was engineered with an *Agrobacterium* PBP (*K_d_* 90 μM; Lager et al., 2006). Perfusion experiments have also been used to investigate transport activities in some studies. FLIPglu–600μΔ and FLIPsuc–90μΔ1 were used in stably transformed Arabidopsis to measure accumulation in roots (Chaudhuri et al., 2008). Results indicate that uptake may be mediated by proton-independent systems. FLIPglu–2μΔ13 was successfully expressed in rice (Zhu et al., 2017). Recently, an affinty series for FLIPsuc was engineered (Sadoine et al., 2021). Next steps will be the use of this suite of sensors to analyze important outstanding questions in physiology.

#### Sensors for amino acids

Amino acids are not only the building blocks of proteins, and precursors for specialized metabolism, but in most plants also the dominant transport forms for organic nitrogen. In addition, amino acids can function as signals in plants, similar as in neurons (Toyota et al., 2018; Castro-Rodríguez et al., 2020). The prototype FLIPE detects glutamate, and proof of concept was achieved by expression and analysis in neuronal cells (Okumoto et al., 2005). More recently FLIPE was implemented in plants (Castro-Rodríguez et al., 2020). Ahmad’s group developed a broad range of amino acid sensors including sensors for leucine (Mohsin et al., 2013), methionine (Mohsin and Ahmad, 2014), lysine (Ameen et al., 2016), isoleucine (Singh et al., 2019), and cysteine (Singh et al., 2020; www.molecular-physiology.hhu.de/resources). The insertion of cpFPs produced iGluSnFr, which was used in animals and plants (Marvin et al., 2013; Toyota et al., 2018). Notably, high-affinity FLIPE version appear to cause “sponge effects” when displayed at cell surface (Castro-Rodríguez et al., 2020). Very similar to what led to the discovery of SWEETs, glutamine transport was investigated in Arabidopsis roots with FLIPQ glutamine sensors (Gruenwald et al., 2012) identifying UMAMIT amino acid uniporter activity in Arabidopsis (Besnard et al., 2016). All these sensors were based on bacterial PBPs, while FLIPW tryptophane sensors were built using dimeric bacterial tryptophane repressors (Kaper et al., 2007). Proof of concept was made by identifying a tryptophane/kynurenine exchange in mammalian cancer cells. FLIPW sensors were originally made as a chassis for engineering auxin sensors, recently realized through further engineering (Herud-Sikimić et al., 2021). While only a few studies have made use of the amino acid sensors in planta, there is now a complete set of sensors for almost all amino acids suitable for plant studies.

#### Hormone sensors

Progress in plant hormone sensing has recently been reviewed (Isoda et al., 2021). Genetically encoded FRET-based sensors are available for auxin, abscisic acid, and gibberellin (Jones et al., 2014; Waadt et al., 2014, 2020; Rizza et al., 2017, 2021; Herud-Sikimić et al., 2021).

#### ROS and redox sensors

Sensors for monitoring ROS and redox changes and their roles in growth, development, pollen germination, symbiosis, and responses to biotic and abiotic stresses have recently been reviewed (Choi et al., 2012; Kostyuk et al., 2020; Müller-Schüssele et al., 2021). In plants, ROS are toxic byproducts but also have important physiological roles. Here, we briefly highlight implementations of H_2_O_2_/redox sensors in various plants and targeting to various locations such as ER, chloroplasts, peroxisomes, and mitochondria, which play important roles in redox physiology and/or ROS homeostasis. In plants, roGFP variants were used to investigate the subcellular redox potentials, while HyPer and modified roGFP sensors such as roGFP2-Orp1 or roGFP2-Tsa2ΔCR were used to investigate H_2_O_2_. Examples are used in Arabidopsis guard cells and roots and systemic ROS and redox signaling (Gilroy et al., 2016; Nietzel et al., 2019; Niemeyer et al., 2020; Fichman and Mittler, 2021; Hipsch et al., 2021; Müller-Schüssele et al., 2021; Ugalde et al., 2021a).

### Steps toward in vivo biochemistry: sensors for transporter activity

Sensors as described above can provide information on steady-state levels, but also rate constants, for example, slopes of concentration changes when the stimulus is added or removed (Okumoto et al., 2008). The slopes provide important information on properties of individual cells or tissues, for example, rate constants and cells capable of taking up particular nutrients or respond to stimuli in planta. Additionally, we need information on in vivo enzyme, transporter, and receptor activities to obtain a full picture, particularly since we often rely only on mRNA levels, in some cases protein levels, but activity is highly regulated in planta. For example, *V_max_* of a protein is determined by the number of molecules transported per second and the active number of proteins at a particular location. Enzymes, receptors, and transporters undergo conformational motions during their reaction cycles (Drew and Boudker, 2016). We hypothesized that the approaches for engineering small molecule sensors can be used to report the activity transporter in vivo. Until now, transporter activity sensors were successfully engineered for ammonium, nitrate, oligopeptide, and glucose (De Michele et al., 2013; Ho and Frommer, 2014; Ast et al., 2017; Park et al., 2020).

*AmTrac and AmTryoshka*. ${\text{NH}}_{4}^{+}$ transporters, highly conserved from Archaea to fungi, plants, and humans, are essential for the uptake of ${\text{NH}}_{4}^{+}$ as a key nitrogen nutrient at least in yeast and plants. Activity is tightly controlled by allosteric feedback circuitry (Loqué et al., 2007; Lanquar et al., 2009; Chen et al., 2020). Screening of insertions of cpGFP into the Arabidopsis ammonium transporter AMT1;3 produced the ${\text{NH}}_{4}^{+}$-activity reporter AmTrac (e.g. AmTrac-100μ; De Michele et al., 2013). Ratiometric AmTryoshka sesnors were engineered by employing the Matryoshka approach (Ast et al., 2017).

*NiTrac and PepTrac*. To monitor nitrate transporter and receptor activity, the dual-affinity nitrate transceptor CHL1/NRT1.1/NPF6.3 was sandwiched between cyan and yellow FPs yielding the NiTrac activity reporter (Ho and Frommer, 2014). When expressed in yeast, NiTrac responded nitrate addition. NiTrac had characteristic biphasic kinetics similar as the native CHL1. Using the same approach, four oligopeptide transport activity sensors were generated in a single step using PTR oligopeptide transporters (Ho and Frommer, 2014). NiTrac and PepTrac also reported interaction with regulatory proteins and effects of mutations on the structure.

*SweetTrac*. The newest addition to this set are reporters of SWEET1 transporter activity, named SweetTrac, which were engineered using a combination of cell sorting and bioinformatics (Park et al., 2020). Expansion to sucrose transporting SWEETs will be useful for exploring posttranslational regulation. The successful engineering of activity sensors for AMT/MEPs, NPF/POTs, and SWEETs indicates that the concept can effectively be implemented for other transporters, receptors, and enzymes.

## Challenges of sensor implementation and use in planta

*Subcellular targeting of sensors.* While a cytosolic sensor does not require a targeting sequence, it is not always easy to visualize analytes and detect them in individual cells in particular since many plant cells carry large vacuoles, squeezing the cytoplasm toward the cell periphery. Nuclear targeting can help in tracking of individual cells (Rizza et al., 2017, 2021). Since the sensors are genetically encoded, targeting by fusion to a signal sequence is straightforward (e.g. nuclear localization signals, ER targeting sequences, organelle targeting sequences, display at the cell surface, or secretion into the cell wall (Figure 2B). ATP and redox sensors were successfully targeted to mitochondria and chloroplasts (De Col et al., 2017; Nietzel et al., 2020). Based on their pK_a_, most sensors will be relatively dim in acidic compartments like vacuole, vesicles, and the cell wall space. Besides targeting to compartments, sensors can be fused to specific proteins such as transporters or targeted to micro- or nanodomains; for example, calcium sensors were targeted to nanodomains by fusion to calcium channels to measure calcium transport activity (Tay et al., 2012; Hochreiter et al., 2015).

### The challenge of determining absolute analyte concentrations

While it is possible to carefully determine the affinity of a sensor for an analyte in vitro, cellular conditions likely impact sensor properties. As mentioned above, pH, redox potential, base levels of the ligand, and other ligands can impact the analysis. In the yeast cytosol, both the glucose sensor with nanomolar affinity as well as the millimolar affinity sensor did not show the expected dynamic range, indicating the presence of base levels at the lower edn and saturation of the transport systems at the upper end (Bermejo et al., 2010). It is therefore recommended, where possible, to try to calibrate the sensor in vivo. For calcium, this can in principle be done with the combination of calcium ionophores and calcium chelators. Effictive calibration was successfully obtained for zinc sensors in Arabidopsis (Lanquar et al., 2014). However, for most other analytes, for example, sugars, there are no calibration tools available. Permeabilization with detergents may help, however, needs to be used with caution, since detergents will also equilibrate ions and pH. The addition of saturating concentrations of the ligand may be useful, especially when using multiple sensors with different affinities, although the ratio between uptake and metabolism may limit cytosolic accumulation. Therefore, extreme caution is needed when trying to interpret differences in fluorescence intensity or intensity ratio between different cells in a tissue or organ. Also here, affinity series provide elegant controls.

### RNA silencing of sensor production in planta

One of the biggest challenges for using genetically encoded sensors in plants has been the impact of gene silencing. Transgene silencing is well known and has been studied extensively. It occurs frequently in Arabidopsis, and a wide array of factors including number and complexity of insertions and vector components may affect silencing (Gelvin, 2017). When the first metabolite sensors were expressed in Arabidopsis, fluorescence was readily detectable in early developmental stages, but was lost or became patchy during further development (Chaudhuri et al., 2011). Gene silencing may possibly be caused by using two GFP genes with almost identical coding sequences in the FRET sensors. However, codon diversified versions of eCFP (named Mars) and Venus (named Aphrodite) did reduce silencing (Deuschle et al., 2006). Sporadically, plants were found that showed sufficient levels of fluorescence to enable quantitative analyses (Chaudhuri et al., 2011). Differences regarding the susceptibility were observed between different constructs and the use of the UBQ10 promoter seemed advantageous over the viral CaMV 35S promoter, yet silencing remained an issue even with alternative promoters. Common to these constructs was the use of strong ubiquitous promoters, which likely amplify silencing. Arabidopsis silencing mutants such as *sgs3*, *rdr6*, or the hypomorphic *sgs2-18* mutants suppress silencing, enabling effective sensor analyses in planta (Adenot et al., 2006; Deuschle et al., 2006). While addressing the issue effectively in Arabidopsis, similar mutants are not necessarily available in other species. Moreover, the silencing mutants are recessive; thus, when using sensors in transgenic lines or T-DNA mutants it is conceivable that additional T-DNAs will enhance silencing (W.-J. Guo and W.B. Frommer, unpublished data). Since *Agrobacterium* strains differ in average number of insertions generated, it may be advantageous to use strains that produce low copy numbers (Zhi et al., 2015). It may be advantageous to avoid strong promoters and deploy screenable markers that are expressed only in a small subset of cells, for example, red FPs driven by the seed coat-specific oleosin promoter (Shimada et al., 2010, 2011; Lampropoulos et al., 2013).

### Sensors in mutants: crosses versus transformation

When combining sensors with mutations, three principal strategies are possible (1) transformation-based introduction into mutant lines; (2) crossing mutant and sensor lines; and (3) transient use of CRISPR-Cas to generate mutations in sensor-expressing lines with the help of haploid induction (Kelliher et al., 2019). Transformation is likely the least preferable approach since sensor levels will be variable, requiring identification of optimal and comparable sensor lines and the risk of introducing new mutations caused by *Agrobacterium* transformation or by somaclonal variation. Crosses would be performed with well-characterized sensor lines. CRISPR/Cas editing can generate mutants that ultimately do not contain transgenes. CRISPR/Cas frequently creates biallelic mutations in the T0 generation, and thus may be another good option to remove potential causes for silencing in other species (Hahn et al., 2020). Haploid induction is possibly the best path for combining mutant lines with sensor constructs in crops.

## Features to consider when optimizing or using fluorescent sensors

Roger Tsien generated the first calcium sensors with the aim of monitoring calcium spiking. In plant biology, many processes occur at a slower time scale, for example, when the critical daylength that triggers flowering is reached, physiological changes likely take hours. The sensors described in this review may not be suitable for slow changes, since photobleaching, focal drift, and other factors that affect baseline can mask signal changes.Transcriptional reporters may be better tools for thse slower responses relative to FP sensors. In some cases, other types of sensors, for example, sensors that recall memories such as CaMPARI may be more suited (Sha et al., 2020).

*Selectivity and specificity*. Selectivity and specificity are two important features to consider when developing or using existing sensors. Selectivity and specificity are determined by the structure and conformational flexibility of a protein (Hedstrom, 2001). The terms are often used interchangeably, but are best used for different aspects: specificity is defined as how restrictive a protein is in its choice of substrate (fewer vs. more substrates). Selectivity is defined by substrate properties and is a quantitative measure of the rate constants for interaction of the protein with the substrate (Butré et al., 2014). Typically, we name a protein based on the activity we measure, however, likely this protein recognizes many thousands of different chemicals, with differing affinities. The glucose/galactose PBP binds glucose and galactose, but likely also many other compounds. The maltose binding protein recognizes maltose, but also malto-oligosaccharides with differing chain length. The glutamate binding protein recognizes glutamate with the highest affinity—relative to other ligands including aspartate, glutamine, and asparagine. As Peracchi put it elegantly (Peracchi, 2018): “Substrate specificity cannot be absolute and is inherently limited. … discrimination between alternative substrates can be relatively low, …Substrate promiscuity helps to fuel an ‘underground’ network of reactions which may represent a basis for further evolution and diversification of metabolism.” A sensor with high selectivity for trehalose, which binds sucrose with low affinity, will in vivo report sucrose dynamics since levels of sucrose exceed trehalose levels. Notably, binding protein selectivity is tested with only few analytes, while the in vivo environment presents a highly complex set of molecules. Rarely, binding protein affinity is suitable for in vivo analyses. Affinity has to be adjusted by mutagenesis and affinity series of the sensors might be required. Mutations in the binding pocket may impact ligand selectivity.

*Orthogonality*. The use of nonnative recognition elements for in vivo applications has obvious advantages, namely low probability to interact with endogenous proteins. For example, carbohydrate and amino acid sensors used in eukaryotes were engineered using bacterial PBPs. In contrast, calcium and plant hormone sensors were based on binding proteins from the target species. This has the advantage that they have suitable affinities when used in the same host. However, endogenous components interact with sensors affecting cellular signaling, or the readout may be affected by interaction of cellular calmodulin binding proteins. Tsien’s lab recognized this issue and used a “bump-and-hole” approach to generate orthogonal calcium sensors less prone to interaction with endogenous factors (Palmer et al., 2006). Alternatively, the impact when using GCAMPs on the physiology of transgenic mice could be eliminated by blocking such interactions as in GCaMP-X (Yang et al., 2018). Similar approaches will likely be useful for other sensors that use endogenous recognition elements.

*Sensitivity, dynamic range*, *and signal-to-background ratio (SBR)*. Sensitivity refers to the ability of a sensor to report minute changes, whereas SBR relates to the signal that can be distinguished from background fluctuations. Sensitivity is a feature of the sensor and has to be defined relative to the analyte levels in the compartment of interest. A sensor with nanomolar affinity is highly sensitive, but cannot report if analyte levels in the compartment are in the millimolar range, because the sensor will be saturated. Starvation of plants or signal difference measurements after addition of saturating levels of the analyte may be useful (Chaudhuri et al., 2011). Sensitivity is maximal around the *K_d_*; in the ideal case, lowest and highest analyte levels fluctuate within the linear detection range of the sensor, emphasizing the need to test affinity series. The detection range of a sensor is defined often by the range in which the binding isotherm has near-linear characteristics, that is, between 10% and 90% saturation; often two orders of magnitude for a FRET sensor with a Hill coefficient of 1. Due to the dramatic intensity changes caused by ligand binding in cFP sensors, the dynamic range can be extended beyond the linear range. SBR depends not only on sensor properties, but importantly on the background fluorescence in the specimen under investigation. In plants, autofluorescence can differ substantially. Handling, pathogen infection or other stress conditions can trigger production of fluorigenic compounds (Donaldson, 2020). Sensor output is typically reported as a change in fluorescence intensity or ratio (Δ*F*/*F*_0_ or Δ*R*/*R*_0_). Relevant sensor properties include brightness (quantum yield, extinction coefficient) and photostability. Photobleaching can be modulated by acquisition intensity and frequency. Thus, for high SBR, higher levels and brighter sensors are advantageous, high sensor levels can impact cellular functions.

*Interference of sensors with cellular functions.* From first principles, one could argue that the higher the sensor level is, the brighter the signal and the better the ability to discern changes in analyte levels or activity. Strong promoters provide high levels, yet besides likely triggering gene silencing, high sensor levels impact physiology, for example, Arabidopsis lines expressing ABA sensors showed ABA-related phenotypes. ABACUS sensor lines were ABA-hypersensitive and behaved similar to ectopical expression of receptors (Jones et al., 2014). Sensitivity corresponded with the affinity for ABA. In contrast, an ABAleon line with exceptional brightness was hyposensitive to ABA, likely acting as a sponge (Waadt et al., 2014). Similar sponging effects were observed in Arabidopsis lines that display FLIPE glutamate sensors at the plasma membrane (Castro-Rodríguez et al., 2020). While sensors are minimally invasive, they can affect cellular functions, either by acting as scavengers or by interacting with other cellular components; essentially posing an “Observer Effect” problem (Buks et al., 1998). In the absence of novel, even less invasive technologies, it will be important perform proper controls. For example, growth curves and flux analysis allowed us to exclude sponging effects in yeast expressing glucose sensors (Bermejo et al., 2010).

*Other potential sources of artifacts*. While ubiquitous promoters should provide more or less equal sensor levels in all cells, and while the output from ratiometric sensors should be independent of sensor levels, in reality emission intensities and ratios vary, and loss of signal loss occurs by scattering. When observed from the side, roots are cylindrical, with a single-cell layer at the surface, but multiple layers at the center. In epifluorescence mode imaging artifacts will likely affect quantification in tissues or cells that change volume. Such artifacts could be evaluated by adding saturating amounts of analyte. When using confocal microscopy for sensor imaging, it is possible that addition of sugars, stresses, or hormones causes changes in the 3D volume and thus a shift to a new *z*-position, thereby providing data from a different region of the cell. Notably, changes in osmolality cause changes in the concentration of all solutes in the cell, as had been observed when monitoring calcium dynamics in mammalian cells exposed to square pulses of glucose (Hou et al., 2009).

## Microscopy and imaging systems

Epifluorescence (also widefield fluorescence) microscopy is a powerful tool for plant quantitative imaging. Plants contain or produce autofluorescent components, causing interference when exciting with ultraviolet or blue light. For multichannel imaging, especially using ratiometric sensors, excitation filter switching speed is an important system parameter (filter wheel or mirror-based devices). Choice of objectives is important. Spectral accuracy is obviously important. Numeric aperture is a unitless measure of the resolution and photon collection efficiency. Objective magnification is not a reliable indicator of resolution, but determines of view, as well as the photon density of the excitation beam at the specimen level, which corresponded to signal brightness and phototoxicity. Brightness, thus sensitivity, is proportional to NA^4^/magnification^2^ (Lichtman and Conchello, 2005). In practice, high brightness can be achieved using high NA (≥1.30) 60× objectives. Whereas dry objectives offer greatest ease of use, high NA objectives require oil, glycerol, or water immersion. Particularly at imaging depths below the outermost cell layer, immersion media and sample should have matching refractive indices (RI) to minimize spherical aberrations. Silicone immersion oil (RI: 1.4) objectives have become popular choices for live-cell imaging. Choice of emission filter specifications is primarily guided by fluorophore emission properties but should also factor in sample autofluorescence. Emission filter switching is accomplished with filter or image-splitting with dichroic mirrors can be used to project multiple channels onto different regions of a single detector, at the expense of reduced photon capture and field of view (FOV). Beamsplitters can also be used to direct channels to multiple detectors, but require proper alignment.

*Choice of camera technology.* Filtered emitted photons are detected by cameras in practically all epifluorescence microscopes. Electron multiplying charge coupled device (EMCCD) cameras were the dominant devices for quantitative sensor imaging; however, by comparison, complementary metal oxide semiconductor (sCMOS) cameras offer advantages in terms of maximal acquisition speed, pixel count, FOV, and price. Each pixel in an sCMOS camera has its own amplifier (commonly a combination of amplifiers, sometimes termed “active-pixel” technology). In current sCMOS architecture, each row of pixels has its own analog-to-digital converter. EMCCD pixel arrays operates as analog devices; all pixels share a common amplifier and digitizer. These differences have important ramifications for quantitative imaging: sCMOS enables higher maximal framerates, but introduces considerable pixel response nonuniformity, manifesting itself as noise in raw images. To compensate, sCMOS cameras are equipped with on-board processors that computationally reduce pixel response nonuniformity. Researchers should therefore be cognizant that data are not “raw” but rather preprocessed.

The SNR compares relative signal and noise. Noise is an inherent feature of devices that detect the signals from sensors. Different noise types arise during digital imaging (Gaussian noise, white noise, Brownian noise, Salt and Pepper Noise, periodic noise, quantization noise, speckle noise, Poisson noise, etc.). In fluorescence microscopy, photon and read noise dominate (Boyat and Joshi, 2015). As for SBR, it is advantageous for reliable quantification to have high signal levels. In EMCCD architectures pixels can be combined or “binned” to improve (SNR), as signals are summed across binned pixels before readout. Pixel binning can also in sCMOS cameras but is fundamentally different because it occurs after readout. Regardless of camera architecture, pixel binning reduces data volume but necessarily comes at the expense of reduced spatial resolution. EMCCD cameras are still the preferred choice for the detection of extremely dim signals or to enable quantitative photon counting. Based on improvements in chip technology sCMOS cameras with quantum efficiencies >90% and read noise below 1.5 e− are available, allowing for combined high sensitivity with fast readout of large FOVs.

### Large FOV imaging

Technological advances in imaging systems generate high demands on data storage and processing. Bit-depth for modern cameras or other detectors is commonly 16-bit. Pixel arrays for EMCCD cameras are 512 × 512 or 1,024 × 1,024, while sCMOS pixel arrays may exceed 2,048 × 2,048 and are limited by data transfer rates. For timelapse imaging with sensors, the resulting data volume, particularly at high framerates, is enormous (typically multiple gigabytes). Compared to epifluorescence imaging, data volume challenges are even more relevant for imaging technologies that make use of optical sectioning and volumetric applications.

Point-scanning confocal microscopy has superior spatial resolution compared to epifluorescence microscopy; however, point-scanning presents disadvantages regarding sensitivity and acquisition speed. Higher illumination intensity can compensate for reduced sensitivity caused by rejection of out-of-focus light, but increases photobleaching and phototoxicity. Increased exposure times can compensate for reduced sensitivity but affect photobleaching and phototoxicity, in addition to lower maximal framerate. High-speed galvanometers and resonant scanners achieve fast framerates. Pinhole adjustment is possible on most systems; and, for quantitative imaging, larger pinholes can improve sensitivity. Point-scanners typically use photomultiplier tubes. avalanche photodiodes or array-based hybrid detectors.

Spinning disk confocal microscopy, a commonly used form of field-scanning confocal microscopy, offers advantages over point-scanning confocal microscopy in terms of FOV, acquisition speed and sensitivity, which is particularly beneficial for timelapse volumetric imaging. By using a rotating array of pinholes, comparatively large FOV can be instantly exposed and photons are collected by cameras, similarly to epifluorescence imaging systems. Optical sectioning along the *z*-axis typically necessitates *z*-stack acquisitions for plant live-cell imaging. Maximal *z*-stack acquisition speed may be important for applications, such as calcium imaging. Optical sectioning also accentuates effects on optical drift, particularly at high magnifications. Drift along the *z*-axis is caused by temperature changes can be compensated devices that adjust the focal position infrared-based measurement of distance between objective and cover glass.

Light-sheet fluorescence microscopy (LSFM), specifically selective plane illumination microscopy, has proven suitable to image subcellular dynamics in tissues over time and complex developmental processes. LSFM has low phototoxicity and high-speed multiview acquisition (Grossmann et al., 2018). Due to the arrangement of the objective lens and the plane illumination lens sample positioning is more complicated and applications are limited to small and transparent specimen. Several currently implemented LSFM configurations are particularly well suited for quantitative imaging of sensor responses. Laser absorbance by cellular structures can create “shadowing” artefacts that can be reduced by pivoting the excitation laser to provide additional access angles to internal structures (Girkin and Carvalho, 2018; Albert-Smet et al., 2019). In any tissue, LSFM does not intrinsically provide any technological advantages in terms of imaging depth, although combination with two-photon excitation lasers or adaptive optics could provide solutions (Truong et al., 2011; Chmielewski et al., 2015; Wolf et al., 2015; Royer et al., 2018).

Total internal reflection (TIRF) microscopy utilizes an evanescent wave of light that acts near the interface of two transparent media, upon illumination at a critical incidence angle. TIRF is restricted to very narrow depths, which makes it particularly well suited for observation of membrane processes (Johnson and Vert, 2017). For quantitative analyses, the strongly nonlinear illumination intensities achieved at increased imaging depth introduce a complicating factor (Oheim et al., 2019). Because of imaging depth limitations, TIRF microscopy for plant imaging applications may be generally restricted to single-cell application, such as calcium imaging of pollen or protonemal tip growth.

Multiphoton excitation microscopy has been widely adopted for improved imaging deep within brain tissue. Infrared photons are absorbed less strongly by brain and most other biological tissue. Under strong excitation using pulsed lasers, fluorophores can absorb two or more long-wavelength photons in a single quantum event and emit a shorter wavelength, higher magnification photon (Denk and Svoboda, 1997; Wang et al., 2020). Multiphoton excitation is a very low probability event, therefore powerful nanosecond-scale pulsed lasers are typically employed. One of the benefits of the low probability of excitation events is that optical section is achieved via the point spread function of the laser excitation, and no pinhole is required (Denk and Svoboda, 1997). Comparatively few studies have employed multiphoton excitation microscopy in plants to date (Cheung et al., 2010; Mizuta et al., 2015).

### FLIM and fluorescence anisotropy imaging

Donor FL correlates negatively with FRET and can be used as a readout for FRET sensors, provided that fluorescence decay curves can be adequately resolved (Datta et al., 2020). Because typical FLs of FPs are in the millisecond range, FL measurements require pulsed illumination sources and rapid time-resolved detectors. Theoretically, hetero-FRET (i.e. FRET between distinct fluorophore species) results in a concomitant reduction in donor fluorescence anisotropy, whereas homo-FRET (i.e. FRET between identical fluorophores) results in increased fluorescence anisotropy due to energy migration. Nonetheless, experimental validation of predicted photophysical behaviors of the rapidly expanding set of available FPs has lagged, and exceptions to theoretical expectations have been noted (Koushik and Vogel, 2008). FL gating can help when dealing with thick or autofluorescent samples by elimination of autofluorescent emission that differs in FL from the FPs.

### OA imaging, three-photon excitation microscopy, and other emerging imaging technologies

OA imaging makes use of the photoacoustic effect (Bell, 1881) and has been used to perform calcium imaging in mammalian brains (Gottschalk et al., 2019). The technology may also be useful in plants to increase imaging penetration depth. Likewise, three-photon excitation microscopy is an active area of investigation and may enable deeper access inside plant structures in the future (Ouzounov et al., 2019). Combinations of the technologies described above provide biologists with powerful new opportunities for quantitative imaging.

### Plate reader-based measurements

Plate reader-based approaches enable multiparametric real-time monitoring of sensor output, for example, for investigating plant redox physiology during development or in stress conditions (Rosenwasser et al., 2010; Wagner et al., 2019; Müller-Schüssele et al., 2021).

## Specimen handling and stimulation

Minimally invasive imaging conditions are prerequisites for measurings signaling in plant cells, for example, calcium transients are elicited during cellular growth or cell-to-cell communication, but also by a range of stress conditions, including mechanical perturbation (Wymer et al., 1997; Monshausen et al., 2008; Monshausen, 2012). Custom-made containers specifically designed to house and protect the specimen have to also position the cell type-of-interest directly at the cover glass—a particular challenge for bulky organs. Home-made chambers for the simultaneous cultivation of Arabidopsis pistils, pollen tubes, and ovules helped uncover communication between male and female gametophytes during double fertilization and cell type-specific calcium signatures in pollen tubes, synergids, egg, and central cells prior to and during gamete fusion (Iwano et al., 2012; Denninger et al., 2014; Hamamura et al., 2014; Ngo et al., 2014).

Chambers or agarose coverings provide protection from drying-out and physical guidance; channels provide paths for roots, pollen tubes or moss protonemata. Microfluidics and microdevice engineering advanced the capabilities of specimen handling (Yanagisawa et al., 2021). Microdevices were specifically developed for sensor imaging in planta. The RootChip-8S, a device featuring parallel microchannels for Arabidopsis roots (Denninger et al., 2019), mounted on an inverted microscope, helped quantifying apical calcium oscillations in growing root hairs and identifying cyclic nucleotide-gated channels with roles in cell integrity signaling during tip growth (Brost et al., 2019). In RootChips, roots are typically analyzed on a horizontal stage. RootChips can, however, also be used vertically with suitable instrumentation (Fendrych et al., 2018). LSFM enabled effective analysis of sensor responses in vertically mounted roots (Costa et al., 2013; Candeo et al., 2017). Customized holders for parallel analysis or multiple seedlings increase throughput (de Luis Balaguer et al., 2016).

*Perfusion devices.* Kinetic studies of stress responses or nutrient uptake require minimal manipulation of specimen and controlled perfusion. A chamber for pulsed perfusion of immobilized Arabidopsis seedlings was developed to record biotic and abiotic stress-induced calcium responses in roots and cotyledons (Krebs et al., 2012; Keinath et al., 2015). Perfusion chambers were also instrumental for measuring sugar transport in roots with FLIPglu glucose sensors (Chaudhuri et al., 2008, 2011). These handmade chambers had the limitation that whole seedlings had to be immobilized prior to analysis. Microfluidics enable on-chip seedling cultivation and precise perfusion with minimal dead volumes. The RootChip, a valved device for 8 or 16 seedlings, enabled square-pulse perfusion with liquid exchange rates fast enough for recording glucose uptake and release kinetics (Grossmann et al., 2011), calibration of eCALWY sensors to quantify cytosolic zinc levels (Lanquar et al., 2014), characterization of ABACUS responses (Jones et al., 2014), and monitoring of stimulus-specific calcium signatures in roots (Keinath et al., 2015). The valve-free RootChip-8S was developed for pulsed perfusion of Arabidopsis roots expressing the gibberellin sensor nlsGPS1 to elucidate gibberellin gradients (Rizza et al., 2021). Microvalves provide rapid liquid exchange rates when time resolution in the range of seconds is needed (Unger et al., 2000); however, simpler devices offer lower fabrication costs, easier use, and higher robustness.

*Localized stimulation*. While the described chambers allow for treatments of whole seedlings or organs, more localized applications are required to address intercellular communication, for example, during perception of and response to local stimuli. Sophisticated tools have been developed for treatments with high spatial precision. Organic electronic ion pumps were emplyed for local auxin application of auxin (Poxson et al., 2017). Three parallel laminar streams were oriented perpendicular to the root in a microfluidic device with two outer streams focusing the central stream to a few µm in roots (Meier et al., 2010). Thereby, auxin was applied to a very narrow zone, stimulating local root hair emergence. Much simpler systems were used to treat agar-covered Arabidopsis root tips via a punched-out hole in the agar with salt solutions (Choi et al., 2014). Laser ablation of single epidermal root cells was applied to simulate nematode feeding, showing that resulting calcium transients remained largely localized (Marhavý et al., 2019). A variant of the RootChip, in which roots are guided through a micropillar array and exposed to dual-laminar flow along the root axis, was used to uncover that salt treatment on one side of the root resulted in a calcium wave that reached the opposite side of the root, while the bacterial peptide flg22 elicited calcium responses only on the treated side (Stanley et al., 2018). While the presented approaches have been primarily designed for the investigation of Arabidopsis roots, numerous devices have been developed for pollen tubes, moss protonemata and, albeit still rare, also in other angiosperms (Yanagisawa et al., 2021). A device specifically designed for the approximately four times thicker root of *Brachypodium* was used to investigate responses to osmotic stress treatments (Khan et al., 2019). Fibrous root systems of rice were allowed to grow into into separate, petaloid chambers with different growth conditions (Chai et al., 2019).

## Choice between different technologies

To determine which technologies are optimal for a certain question, various criteria such as SNR, signal changes and sensitivity of the sensors, binding affinity, kinetics, undesired artefacts and buffering capacity, low invasive, spatial and temporal resolution, multicolor approaches, dynamic range, time resolution, spatial resolution, FOV, throughput, and dimensionality should be considered (Box 2).

Box 2. Choice between different technologies.

*Sensor design:* Depending on the biological question temporal and spatial aspects are required (Figure, A). Principally one needs to decide whether transcriptional reporters or fluorescent sensors may be more suitable for the biological question. Important criteria for choice of sensors design include orthogonality, dynamic range, affinity close to the steady-state concentrations, which rely on the choice of the sensory domain. The choice of the binding protein influences design possibilities (e.g. 3D structures required for easy design of cpFP-based sensors and to predict conformational rearrangements in cpFP-or FRET-based sensors). The choice of the sensor design to a certain extent relies on the combination with other technologies. For example, the uses of next generation sequencing, high-throughput screening technologies (e.g. FACS), iterative directed evolution might be needed for the first successful design and for optimization. The choice of the FP can be important (e.g. pK_a_, spectrum distinct from autofluorescence). The imaging technology (e.g. choice of excitation/emission parameters to avoid autofluorescence) and multiplexing have be considered for sensor design.

*Imaging system:* For selection of an imaging platform, tradeoffs exist among spatial resolution, temporal resolution, and sensitivity (Figure, B). Sensitivity is critical for quantitative sensor imaging experiments and is greatest for epifluorescence microscopy, which also offers highest temporal resolution. Confocal fluorescence microscopy offers greater spatial resolution at the expense of sensitivity and speed. Light sheet fluorescence microscopy offers sensitivity and temporal resolution that can rival epifluorescence while offering superior spatial resolution similar to confocal systems. Optical sectioning techniques are more susceptible to *z*-axis artefacts introduced by sample movement or changes in cell volume (e.g. due to osmotic effects) relative to epifluorescence microscopy. It is advisable to weigh the relative importance of specific parameters before selecting the imaging platform. If spatial resolution and dimensionality are highly relevant, technologies that combine optical sectioning with sensitivity and speed suitable for quantitative sensors imaging such as multipoint confocal microscopes with greater FOV and acquisition speed are available (e.g. spinning disk confocal microscopes).

*Specimen handling:* Used are typically: (1) agar-based systems; (2) perfusion chambers; and (3) microfluidics. Choice depends on the needs of sample preservation, treatment control, and throughput (Figure, C).
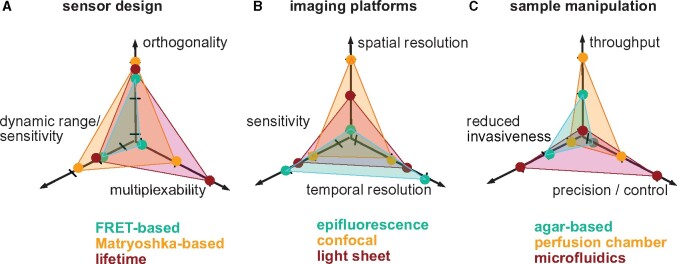

**Figure** Choice of technologies. Some criteria for (A) sensor design, (B) imaging technology, and (C) sample manipulation; axes provide parameters. Colored dots: relative performance scores for sensors, fluorescence microscopy and specimen handling.

## Concluding remarks

As of today, a cornucopia of sensors for ions, metabolites, redox potential, biophysical processes as well as a first set of activity sensors has been engineered (www.molecular-physiology.hhu.de/resources). Many require further optimization before they can be used effectively in vivo. In parallel, quantitative imaging technologies evolve rapidly. In combination, there are new options to gather detailed quantitative cellular and subcellular data with high temporal resolution for in vivo biochemistry. There are challenges and pitfalls, but also solutions, and as long as no revolutionary new technology springs up, sensors provide a powerful tool set to explore the biology of plants with unique perspectives based on the expansion of possibilities in sensing domains, FPs, and quantitative imaging technologies (see “Outstanding questions”; Box 3; de Felipe et al., 2006; Fosque et al., 2015; Qian et al., 2019; Arce-Molina et al., 2020).

**Outstanding questions**

- Can sensor design and optimization be accelerated by using improved FPs, structural information, and machine learning high-throughput screening?
- Can the approaches used for designing transporter activity sensors be applied to enzymes as well?
- A wide range of sensors and activity reporters is now available, so how efficiently can these tools be used in planta?
- Can the use of FL sensors be generalized in a way to overcome drawbacks encountered in complex quantitative imaging in plants (e.g. multiplexing)? How can imaging systems and sensor design approaches be further improved for rendering FLIM sensors more accessible?
- How can a better understanding of the causes of sensor silencing help in addressing major obstacles of the implementation of sensors in plants?

Box 3. Future improvement of sensors.

Some genetically encoded fluorescent sensors, especially for calcium, have been optimized for almost 25 years. Yet, there is still a large window of improvement in sensor design and quantitative imaging in vivo by expanding the panel of sensory domains, colors, imaging systems and readouts. Many sensors for metabolites have been designed by using bacterial PBPs. Recently, sensors using operon repressor family transcription factor or other sensory domain belonging to families of protein which can bind a broad spectrum of molecules have emerged (Arce-Molina et al., 2020). While a cornucopia of FPs is available now, there is room for further improvement, improved quantum yield, maturation, resistance to photobleaching, lifetimes, optimal matching for Matryoshka approaches, multiplexing, etc. Examples include the near infrared NIR-GECO1 calcium sensors; particularly suited for multiparameter imaging (Qian et al., 2019). The Zhang lab implemented a suite of tools for multiplexing and simultaneous recording of processes in animal systems making use of differential subcellular targeting and FLIM sensors (Greenwald et al., 2018). Recently, 2-in-1 genetically encoded fluorescence indicators (GEFI) carrying two GEFI separated by 14-amino acid linker or the “self-cleaving” 22-amino acid P2A linker have been used in multiparametric imaging analysis (de Felipe et al., 2006).

## Funding

This research was supported by the Deutsche Forschungsgemeinschaft (DFG, German Research Foundation) under Germany’s Excellence Strategy—EXC-2048/1—Project ID 390686111 and SFB 1208—Project-ID 267205415, the Alexander von Humboldt Professorship, the European Research Council (ERC) under the European Union’s Horizon 2020 research and innovation program (grant agreement no. 951292, Sympore), and a Japan Society for the Promotion of Science (JSPS) grant (19H000932) to W.B.F., a Heisenberg Professorship (GR4559_4-1) and a DFG research grant (GR4559_5-1) to G.G., by the Human Frontier Science Program to M.N., by the Ministry of Science and Technology (MOST-109-2311-B-001-021 and MOST-110-2923-B-001-002-MY3) and Agricultural Biotechnology Research Center (ABRC) of Academia Sinica, Taiwan to C.H.H. M.N. was supported by a grant from the Japan Society for the Promotion of Science (20K21424) and ITbM (World Premier International Research Center Initiative, WPI, Japan).

*Conflict of interest statement*. None declared.

## Notes

Concept: W.B.F. and C.H.H. M.S., Y.I., T.J.K., M.M.W., M.N., G.G., W.B.F., and C.H.H. contributed to the writing of the manuscript. Figures were developed by M.S., T.J.K., and G.G.

The author responsible for distribution of materials integral to the findings presented in this article in accordance with the policy described in the Instructions for Authors (https://academic.oup.com/plphys/pages/general-instructions) is Cheng-Hsun Ho (zcybele3@sinica.edu.tw).
